# An Indicator Based on Spatial Coordinate Information for Assessing the Capability for Dynamic Machining Performance of Five-Axis Flank Milling

**DOI:** 10.3390/s24227229

**Published:** 2024-11-12

**Authors:** Chenglin Yao, Gaiyun He, Yicun Sang, Chen Yue, Yichen Yan, Sitong Wang

**Affiliations:** 1 Key Laboratory of Mechanism Theory and Equipment Design of Ministry of Education, Tianjin University, Tianjin 300072, China; yaocl@tju.edu.cn (C.Y.); yuechen0327@163.com (C.Y.); ycyan@tju.edu.cn (Y.Y.); wst0511@tju.edu.cn (S.W.); 2 Engineering Training Center, Tianjin University of Technology and Education, Tianjin 300072, China; sangyicun@tju.edu.cn

**Keywords:** spatial coordinate information, error mutual moment, five-axis machine tool, dynamic machining performance, flank milling

## Abstract

As a spatial coordinate sensor, the touch-trigger on-machine probe is a key equipment in manufacturing that ensures machining quality, and it has played an important role in five-axis flank milling. However, in flank milling, the utilization of the deviation as a conventional indicator for quality assessment of the machining performance is incomprehensive without considering the characteristics of the machining method. In this paper, the error mutual moment is introduced as an indicator to assess the capability for dynamic machining performance of the machine tool in flank milling based on the spatial coordinate information of the touch-trigger on-machine probe considering the characteristic of the error distribution of the flank milling. Experiments are carried out to validate the advantages of the error mutual moment to assess the capability for dynamic machining performance compared with the deviation. Results show that the error mutual moment shows more significant discrepancies than the deviation in assessing the capability for dynamic machining performance of flank milling. The error mutual moment has the potential to be applied as a quality assessment sensor.

## 1. Introduction

As a common part of a spatial coordinate sensor, the touch-trigger on-machine probe is the most essential component of the on-machine verification system. In conjunction with the numerical control system of the machine tool, the on-machine verification system can generate the three-dimensional coordinates of the corresponding points, which is capable of replacing the function previously served by the CMM (coordinates measurement machine). Furthermore, the deviation is selected as an indicator for quality assessment of the machining performance regardless of how the formed surface is machined. Furthermore, the interaction between the surface forming method and the error distribution of the formed surfaces enables a more comprehensive inversion of the factors that affect the generation of errors.

Five-axis flank milling, owing to its advantage of a high material removal rate, has become widespread in manufacturing, especially in aerospace and shipbuilding fields, which necessitates higher requirement for the balance of accuracy and efficiency. Taking integral impellers or blades as an example, these workpieces possess added value and high accuracy; thus, the manufacturing process of the workpiece must be rigorous, preventing out-of-tolerance scraps. Yu et al. [1] introduced an R offset tool axis error calculation method, and they derived a new geometric model of tool axis vector for flank milling non-developable ruled surfaces and machining error exact analytical solutions. Moreover, global optimization is concerned more on the relationship between the designed envelope and the machining envelope. Zhang et al. [2] established an optimization model considering machine tool kinematics; complex spatial engagements were represented on a group of planes, and the machining region of each path was improved by adjusting the contact points and tool orientations. González et al. [3] invented a special device to machine the integral blade rotors. Urbikain et al. [4] presented a time-domain model to predict the surface roughness machined by flank milling.

Recently, several studies were carried out in order to obtain the geometric quality of the formed surface, which was used for the application expansion based on sensor information. Li et al. [5] and Unai et al. [6] reviewed the improvement and the applications of the on-machine verification technologies in machining. Wan et al. [7] introduced a method for inspection path generation according to the structure of the measured surface. Tan et al. [8] proposed a sampling strategy for the measured points based on the geometric feature of the measured surface. As a spatial coordinate sensor, the study of inspection accuracy is unavoidable. Unai et al. [9] proposed a method to assess the uncertainty budget of the inspection system. Zhao [10], Michal [11], Cai [12], Ahn [13], and Li et al. [14] focused more on the pre-travel error. It is not possible to prevent this error due to the fundamental properties of on-machine measurements. Therefore, the researchers performed several experiments to analyze the characteristics of the error and calibrate the location information. It is widely acknowledged that an increased amount of measurement points facilitates a more precise assessment of the geometric quality of the surface under inspection. Nevertheless, the great amount of measurement points leads a low efficiency of the assessment. To solve these problems, Obeidat et al. and Rajamohan et al. [15] proposed sampling methods based on curvature of the measured surface. Moreover, Sang et al. [16] proposed an adaptive method to sample the key measurement point, providing a comprehensive response to the geometric quality of the measured surface. Since the spatial coordinate information output from the on-machine verification system contains a lot of random errors, a number of data processing methods have been employed for the purpose of separation of the errors. For example, Chen et al. [17,18,19] proposed a spatial statistical algorithm, EMD (empirical mode decomposition) algorithm, and CEEMDAN (complete ensemble empirical mode decomposition with adaptive noise) algorithm to decompose systematic and random errors based on measuring data, and compensated the systematic errors by modifying NC codes.

It is challenging to achieve a machined surface that precisely matches its design specifications using a cylindrical cutter [20]. Researchers have developed various methods to improve the conformity of the machined surface with the cutter’s envelope surface. Liu [21] proposed the classic SPO (single-point-offset) and DPO (double-point-offset) methods to avoid gouges and interference. In addition, Redonnet et al. [22] proposed a mathematical formula to describe the interference between a ruled surface and a cutter. Due to the complexity in solving the formula, Bedi et al. [23], Menzel et al. [24], and Guan et al. [25] proposed several methods to simplify the calculations. Sun et al. [26] proposed a HATPG (high-accuracy tool path generation) method by designing the instantaneous envelope surface of each planned cutter location. All the aforementioned methods belong to the local toolpath optimization algorithm. Thus, the essential condition for the implementation of the toolpath local optimization algorithm is that the theoretical contact line and the actual contact line are located in the same plane. Global toolpath optimization algorithms are increasingly being utilized to minimize error throughout the entire processing. Lartigue et al. [27] proposed a kinematics method to calculate, assess, and correct the toolpath. Zhu et al. [28] proposed a global toolpath optimization algorithm based on the minimum zone criterion recommended by ANSI [29] and ISO [30] standards for tolerance evaluation. The author proposed a DPM (double-point-mirror) method, which has been published in the literature [31]. All of the methods are applied on the basis that the line of the error distribution is coplanar with the theoretical contact line.

In five-axis flank milling for complex surfaces, the variations in motion among each axis are not same, especially between the translational axes and rotational axes. This phenomenon is often observed in areas where the feedrate varies dramatically. Consequently, the machining quality in these areas deteriorates significantly. Typically, this phenomenon can be attributed to the poor dynamic machining performance of the surface geometric quality. To access the machining performance of the machine tool, a part with a special shape was designed elaborately. The S-shaped test piece was first proposed by Mou et al. [32] to replace the part described in NAS (national aerospace standard) 979 [33], and the shape has been developed by many researchers [34,35,36,37] in the following years. Finally, the S-shaped test piece was adopted as an informative annex of ISO 10791-7 [38] in 2020. Ihara et al. [39,40] designed an 8-shaped test considering the different performance of CAM software during the machining. Without exception, the deviation under the same machining parameters is selected as the indicator to assess the machining performance. However, the deviation is influenced by a variety of factors, the sources and proportions of which remain unclear. Thus, the utilization of the deviation as a conventional indicator for quality assessment of the machining performance is incomprehensive without considering the characteristics of the machining method. Consequently, there are no indicators that can be universally accepted as a target for assessing the capability for the dynamic machining performance for flank milling.

Herein, the error mutual moment is proposed as an indicator to assess the capability for dynamic machining performance of the machine tool based on the spatial coordinate information of the touch-trigger on-machine probe, which has the potential to be applied as a quality assessment sensor. In addition, through considering the characteristic of the error distribution of the flank milling, based on the spatial coordinate information of the touch-trigger on-machine probe, this paper presents a mathematical derivation of the error mutual moment, employing the principles of space geometry, based on the location information given by the probe. Subsequently, a comparative experiment is carried out to valid the effectiveness of the error mutual moment. Additionally, the experimental results demonstrate that the error mutual moments display more pronounced discrepancies than the deviations when assessing the capability for dynamic machining performance of machine tools.

## 2. Methods

**Figure 1:**
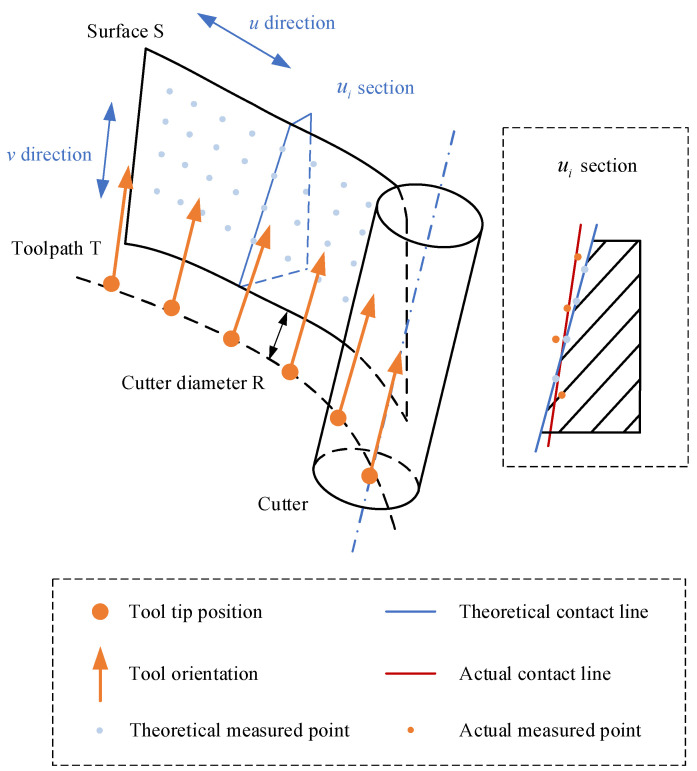
The distribution of measuring points in each $u_{i}$ section.

**Figure 2:**
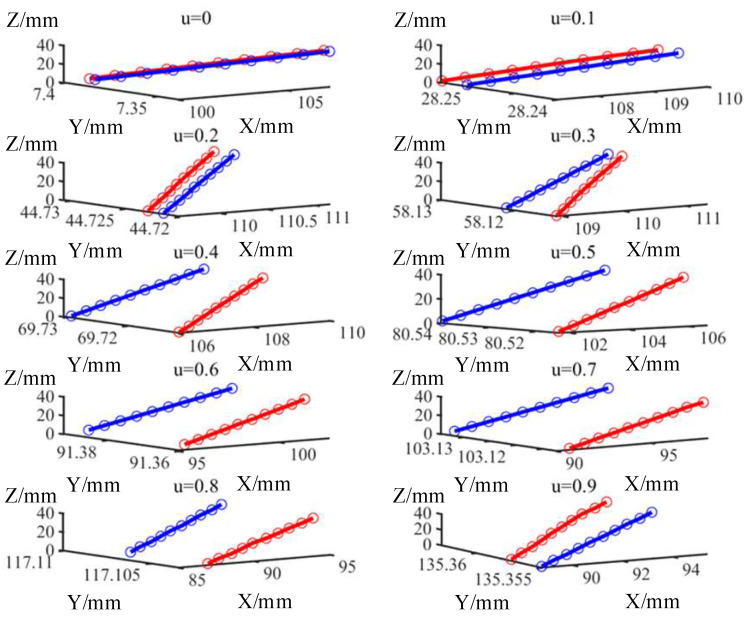
The illustrate of the two lines.

There must be a difference between the theoretical control trajectory of the CNC system and the actual motion trajectory during 5-axis linkage machining due to the manufacturing errors among each manufacturing process and the variations in dynamic stiffness among each component of the machine tool. This difference is the direct reason for errors on the machined surface. For five-axis flank machining, although the error is a result of a number of sources, which are not independent and difficult to be spilt, it can be illustrated as Figure 1. For the surfaces formed by flank milling, the error distribution is nearly linear in the single *u* section [31]. The points in the single *u* section can be fitted by two lines, which is shown in Figure 2.

The traditional method for error assessment is determined by the point-to-point deviation, which is based on the spatial coordinate information of the theoretical point and actual measuring point. Typically, the traditional method of error modelling involves an analysis of the impact of the error source on the tool, with a subsequent development of the relationship between the error source and the error. Therefore, for flank milling, the traditional error analysis method employed does not make adequate use of the whole spatial coordinate information, without considering the coplanarity between the theoretical contact line and the actual contact line during the machining. In this section of the paper, an indicator is proposed to assess the capability of the dynamic machining performance by analyzing the spatial interrelationship between the theoretical contact line and the actual contact line in each $u_{i}$ section. This indicator assesses the capability of dynamic machining performance of a machine tool by employing a methodology that determines the degree of non-coplanarity between two spatial straight lines.

### 2.1. Relationship Between Two Straight Lines in Space

**Figure 3:**
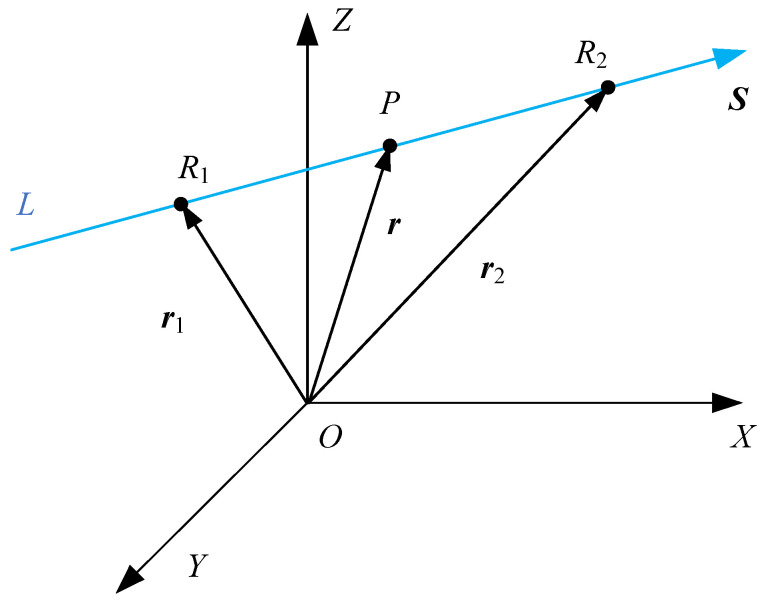
Description of a spatial line.

It is well known that a line in space is defined by two points. As shown in Figure 3, assuming that the straight-line *L* is determined by point *R*_1_ and point *R*_2_, $r_{1}=\vec{OR_{1}}$, $r_{2}=\vec{OR_{2}}$, $r=\vec{OP}$, $S=\vec{R_{1}R_{2}}$. For any point *P* on line *L*, Equation (1) is valid.

(1) $$\left(r-r_{1}\right)\times S=0$$

The equation also can be expressed as:

(2) $$r\times S=S_{0}$$

where $S_{0}$ is called the linear moment of S with respect to the origin *O*. In other words, a straight-line *L* can be determined by the given S and $S_{0}$. In screw theory, $\left(S;S_{0}\right)$ is called the Plücker coordinates of the line *L*.

**Figure 4:**
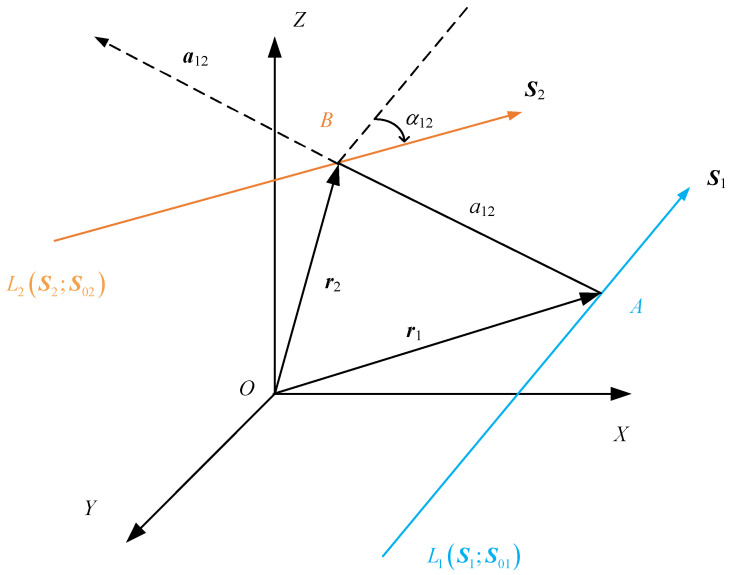
Mutual moment of two straight lines.

As shown in Figure 4, assuming that there are two different straight lines $L_{1}\left(S_{1};S_{01}\right)$ and $L_{2}\left(S_{2};S_{02}\right)$, Equation (3) can be obtained according to Equation (2). In Figure 4, points *A* and *B* are the projections of the common perpendicular line between *L*_1_ and *L*_2_, $\alpha_{12}$ is the twist angle of the two lines, $a_{12}$ is the perpendicular distance between two lines, $a_{12}a_{12}$ is the vector of the common perpendicular line, and $a_{12}$ is the unit vector of the common perpendicular line.

(3) $$\left\{\begin{array}{l} r_{1}\times S_{1}=S_{01}\\ r_{2}\times S_{2}=S_{02} \end{array}\right.$$

### 2.2. The Description of Mutual Moment

The product of the linear moment of $S_{2}$ with respect to point *A* and $S_{1}$ is called the moment of the line *L*_2_ about *L*_1_, and it can be expressed as $a_{12}a_{12}\times S_{2}\cdot S_{1}$. Similarly, the product of the linear moment of $S_{1}$ with respect to point *B* and $S_{2}$ is called the moment of the line *L*_1_ about *L*_2_, and it can be expressed as $a_{12}a_{21}\times S_{1}\cdot S_{2}$. Obviously, $a_{12}a_{12}\times S_{2}\cdot S_{1}=a_{12}a_{21}\times S_{1}\cdot S_{2}$.

In screw theory, these two moments are called the mutual moment of the two lines, which is marked as Μm, and it can be expressed as:

(4) $$Μm=a_{12}a_{12}\times S_{2}\cdot S_{1}$$

since $a_{12}a_{12}=r_{1}-r_{2}$, the equation can be expanded as:

(5) $$Mm=S_{1}\cdot S_{02}+S_{2}\cdot S_{01}$$

Equation (5) is called the general expression for mutual moments. When $S_{1}$ and $S_{2}$ are unit vectors, $S_{2}\times S_{1}=-a_{12}\sin\alpha_{12}$ is valid, and Μm can be expressed as:

(6) $$\begin{array}{ll} Mm & =a_{12}a_{12}\times S_{2}\cdot S_{1}\\ & =a_{12}a_{12}\cdot S_{2}\times S_{1}\\ & =a_{12}a_{12}\cdot \left(-a_{12}sin\alpha_{12}\right)\\ & =-a_{12}sin\alpha_{12} \end{array}$$

The following conclusions can be drawn from Equation (6):

(1) The mutual moment Μm is only related to the distance $a_{12}$ and the twist angle $\alpha_{12}$ and is independent of the choice of origin *O*. In other words, the mutual moment is independent of the choice of the coordinate system.
(2) If two lines are parallel to each other, or if two lines cross at infinity, which means that $a_{12}=0$ at these two conditions, then mutual moment Μm of the two lines is equal to zero. If two straight lines cross each other, which means that $\alpha_{12}=0$ at this condition, then the mutual moment Μm of the two lines is equal to zero. Therefore, if two spatial lines are coplanar, then the mutual moment Μm of the two lines is equal to zero.
(3) If the two direction vectors are not unit vectors, $S_{1}=\lambda_{1}{S^{\prime }}_{1}$ or $S_{2}=\lambda_{2}{S^{\prime }}_{2}$, where ${S^{\prime }}_{1}$ and ${S^{\prime }}_{2}$ are unit vectors of the lines, in accordance with the principles of the vector cross product algorithm, the mutual moment Μm can be expressed as $Mm=-\lambda_{1}\lambda_{2}a_{12}sin\alpha_{12}$.
(4) The smaller the absolute value of the mutual moment, the greater the degree of coplanarity between the two straight lines.

### 2.3. The Presentation of Error Mutual Moment

Generally, the theoretical coordinate of the measurement point is known in advance in the process of acquiring spatial coordinate information. Assuming that the group of coordinates of the theoretical measured points for a surface can be expressed as:

(7) $$P^{T}=\left[\begin{array}{c} P_{u_{1},v_{1}}^{T}\\ P_{u_{1},v_{2}}^{T}\\ \vdots \\ P_{u_{i},v_{j}}^{T}\\ \vdots \\ P_{u_{M},v_{N}}^{T} \end{array}\right]=\left[\begin{array}{ccc} x_{u_{1},v_{1}}^{T} & y_{u_{1},v_{1}}^{T} & z_{u_{1},v_{1}}^{T}\\ x_{u_{1},v_{2}}^{T} & y_{u_{1},v_{2}}^{T} & z_{u_{1},v_{2}}^{T}\\ \vdots & \vdots & \vdots \\ x_{u_{i},v_{j}}^{T} & y_{u_{i},v_{j}}^{T} & z_{u_{i},v_{j}}^{T}\\ \vdots & \vdots & \vdots \\ x_{u_{M},v_{N}}^{T} & y_{u_{M},v_{N}}^{T} & z_{u_{M},v_{N}}^{T} \end{array}\right],\;u_{i}\in \left[0,1\right],\;v_{j}\in \left[0,1\right],\;i=1,2,\cdots M,\;j=1,2,\cdots N$$

where $P_{u_{i},v_{j}}^{T}$ is the theoretical point in $\left(u_{i},v_{j}\right)$ at the measurement surface, of which the coordinate is $\left(x_{u_{i},v_{j}}^{T},y_{u_{i},v_{j}}^{T},z_{u_{i},v_{j}}^{T}\right)$, and *M* and *N* are the number of measurement points in *u* and *v* direction, respectively. Correspondingly, assuming that the group of coordinates of the actual measured points for a surface can be expressed as:

(8) $$P^{M}=\left[\begin{array}{c} P_{u_{1},v_{1}}^{M}\\ P_{u_{1},v_{2}}^{M}\\ \vdots \\ P_{u_{i},v_{j}}^{M}\\ \vdots \\ P_{u_{M},v_{N}}^{M} \end{array}\right]=\left[\begin{array}{ccc} x_{u_{1},v_{1}}^{M} & y_{u_{1},v_{1}}^{M} & z_{u_{1},v_{1}}^{M}\\ x_{u_{1},v_{2}}^{M} & y_{u_{1},v_{2}}^{M} & z_{u_{1},v_{2}}^{M}\\ \vdots & \vdots & \vdots \\ x_{u_{i},v_{j}}^{M} & y_{u_{i},v_{j}}^{M} & z_{u_{i},v_{j}}^{M}\\ \vdots & \vdots & \vdots \\ x_{u_{M},v_{N}}^{M} & y_{u_{M},v_{N}}^{M} & z_{u_{M},v_{N}}^{M} \end{array}\right],\;u_{i}\in \left[0,1\right],\;v_{j}\in \left[0,1\right],\;i=1,2,\cdots M,\;j=1,2,\cdots N$$

where $P_{u_{i},v_{j}}^{T}$ is the actual point in $\left(u_{i},v_{j}\right)$ at the measurement surface, of which the coordinate is $\left(x_{u_{i},v_{j}}^{M},y_{u_{i},v_{j}}^{M},z_{u_{i},v_{j}}^{M}\right)$.

In alignment with the least squares principle, the theoretical measurement points $P_{u_{i},v_{j}}^{T}\left(j=1,2,\cdots N\right)$ and the theoretical measurement points $P_{u_{i},v_{j}}^{M}\left(j=1,2,\cdots N\right)$ in $u_{i}$ section can be fitted by a spatial line in space, which represent the theoretical contact line $L_{u_{i}}^{G}$ and actual contact line $L_{u_{i}}^{C}$, respectively.

**Figure 5:**
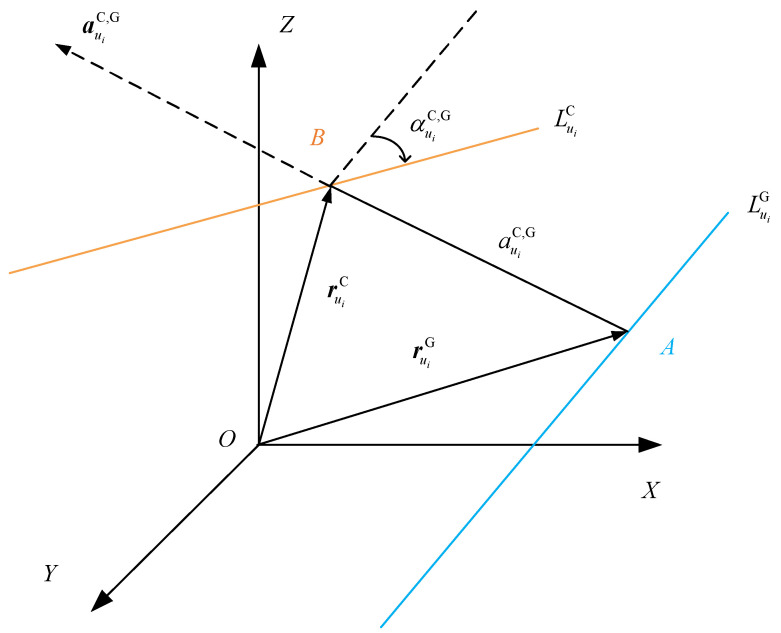
Mutual moment of actual contact line and theoretical contact line.

As shown in Figure 5, the blue line is the fitted theoretical contact line $L_{u_{i}}^{G}$, the orange line is the fitted actual contact line $L_{u_{i}}^{C}$, $\alpha_{u_{i}}^{C,G}$ is the twist angle between the two lines, $a_{u_{i}}^{C,G}$ is the perpendicular distance between the common perpendiculars of the two lines, and $a_{u_{i}}^{C,G}$ is the direction vector of the common perpendiculars of the two lines.

Based on Equation (6), the sign of the mutual moment Μm denotes the relationship between the spatial position of the two straight lines, and the sign is opposite to that which results from the right-hand spiral rule after the two direction vectors have been multiplied. Accordingly, in this paper, the absolute value of the mutual moment between the actual contact line and the theoretical contact line within a given section is defined as the error mutual moment, with both direction vectors represented as unit vectors in the calculation, which is employed as an indicator for assessing the degree of straight-line coplanarity. Therefore, the error mutual moment Μe can be expressed as:

(9) $$Me\left(u_{i}\right)=\left|Mm\left(u_{i}\right)\right|=a_{u_{i}}^{C,G}sin\alpha_{u_{i}}^{C,G},\;u_{i}\in \left[0,1\right]$$

**Figure 6:**
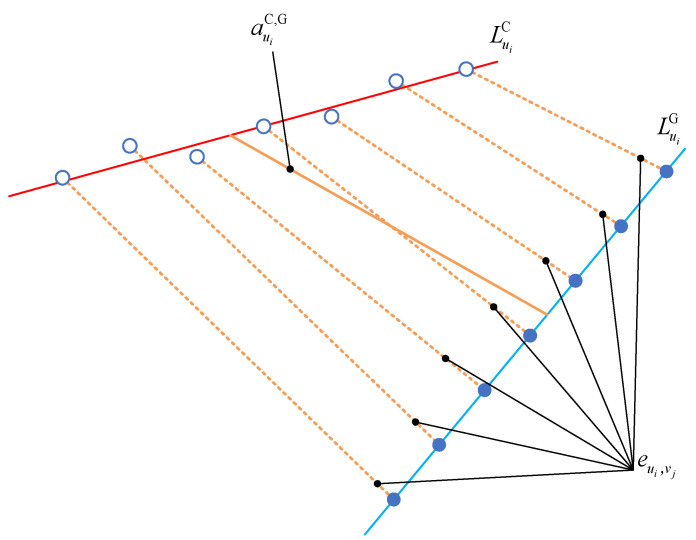
Relationship between error mutual moment and deviation.

In accordance with the geometrical properties governing the mutual moments, the error mutual moments are characterized by the following properties:

(1) The value of the mutual moment of error is independent of the selected coordinatization system. In the measuring process, the selection of the measuring coordinate system is often based on the geometric relationship between the forming surfaces of the workpiece. In the manufacturing process, the selection of the machining coordinate system is often based on the shape characteristics of the blank, and the selection of the workpiece coordinate system is often based on the structural function of the workpiece. Therefore, the determinations of zero point of each coordinate system are not aligned. In the calculation of the error mutual moment, it is not necessary to convert the coordinates of the measuring points of the component obtained in the measuring coordinate system to those of the workpiece coordinate system.
(2) The smaller the value of the error mutual moment, the higher the degree of coplanarity between the two lines in this section. From Equation (9), the value of error mutual moment is determined by $\alpha_{u_{i}}^{C,G}$ and $a_{u_{i}}^{C,G}$; thus, the error mutual moment is a consequence of the interaction between two factors: the perpendicular distance and the twist angle of the two lines. Comprehensively, the smaller the value of the error mutual moment, the higher the degree of coplanarity between the two lines.
(3) Similar to the deviation, the mutual moment of error is determined by several factors during the machining. The qualitative expression for the error mutual moment can be expressed as:
  (10) $$Me=f\left(C,M,\cdot \cdot \cdot \right)$$
  where *f* is the qualitative expression for the error mutual moment, **C** is the matrix of cutting parameters during machining, including but not limited to cutter parameters, the cutter eccentricity, or the error of the fixture. **M** is the matrix of the machine tool evolving machining, including but not limited to the topological structure, choice of the interpolation algorithm, or dynamic stiffness of each axis of the machine tool. In existing evaluations of the geometric quality of surfaces, the deviation $e_{u_{i},v_{j}}$ between two points is similar to the perpendicular distance $a_{u_{i}}^{C,G}$ between the perpendiculars of two straight lines since it refers to the degree of deviation of the actual points (lines) from the theoretical points (lines), i.e., the two indicators are of the same order of magnitude. It can thus be surmised that the deviation between the two points is affected by the same factors as the perpendicular distance between the two straight common perpendiculars. In other words, the deviation is related to the real-time machining parameters and the choice of machine tool. In this paper, $\alpha_{u_{i}}^{C,G}$ is defined as the twist angle between the actual contact line and the theoretical contact line; thus, $\alpha_{u_{i}}^{C,G}$ is related to dynamic lag of the tool orientation during machining, and it must be related to the choice of machine tool.
(4) The utilization of error mutual moment provides a more comprehensive information of the CNC machine’s capability for dynamic machining performance. Especially, the error mutual moment has more advantages on assessing the capability for dynamic machining performance compared with the deviation. Figure 6 describes the relationship between error mutual moment and deviation. $L_{u_{i}}^{C}$ and $L_{u_{i}}^{G}$ denote the fitted actual and theoretical contact line, respectively. The blue hollow points are theoretical points, and the blue solid points are actual measurement points. The orange solid line is the perpendicular distance between the two lines, and the orange dotted lines are the deviations between two corresponding points. Based on the law of spatial geometric relationship, it is known as:
  (11) $$a_{u_{i}}^{C,G}\leq \frac{1}{N}\cdot \sum_{j=1}^{N}e_{u_{i},v_{j}}$$
  The equality sign is satisfied if and only if the two lines are coplanar; and even if this situation occurs, $\alpha_{u_{i}}^{C,G}=0$, $Me\left(u_{i}\right)=0$. Accordingly, the value of the error mutual moment provides information regarding both the distance information and angular information between the actual and theoretical contact lines in this section.

## 3. Experiment

A qualitative experiment is carried out to valid the effectiveness of the properties of the error mutual moment listed in the last section. The machining test is a typical acceptable method to assess the machining performance of a CNC machine tool.

### 3.1. Setup of the Experiment

In this experiment, three workpieces are machined by three different types of machine tools with completely different topological structures, respectively. Measuring and calculating the maximum error mutual moment of the workpiece allow for qualitatively assessing the machining performance of different machine tools.

**Figure 7:**
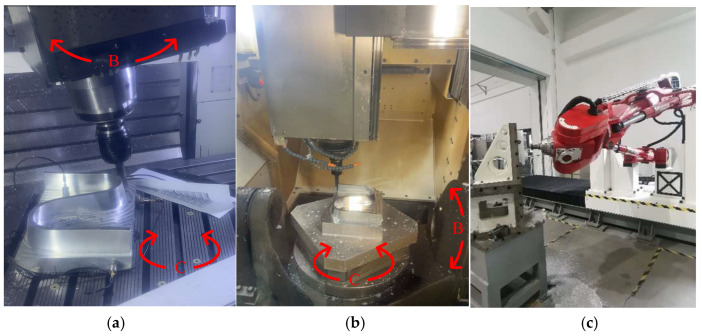
The selected machine tools. (**a**) DMG machining center. (**b**) GF machining center. (**c**) Hybrid robot.

As depicted in Figure 6, three machine tools with entirely different topological structures were utilized in this experiment. Figure 7a shows a DMG vertical machining center with a tilting head (B axis) and a rotary table (C axis). Figure 7b shows a GF cradle-type machining center with two rotary axes (B and C axes) on the workpiece side. Figure 7c shows a TriMule-800 hybrid robot, which was described in detail in ref. [41]. The basic information of the three machine tools is listed in Table 1, Table 2 and Table 3, respectively.

The S-shaped test piece, a standard part used to test the machining precision of five-axis CNC machine tools [42], which serves as the experimental workpiece, was chosen according to the specifications outlined in ISO 10791-7:2020 [38] The 3D model and the cutting parameters are listed in Table 4.

**Figure 8:**
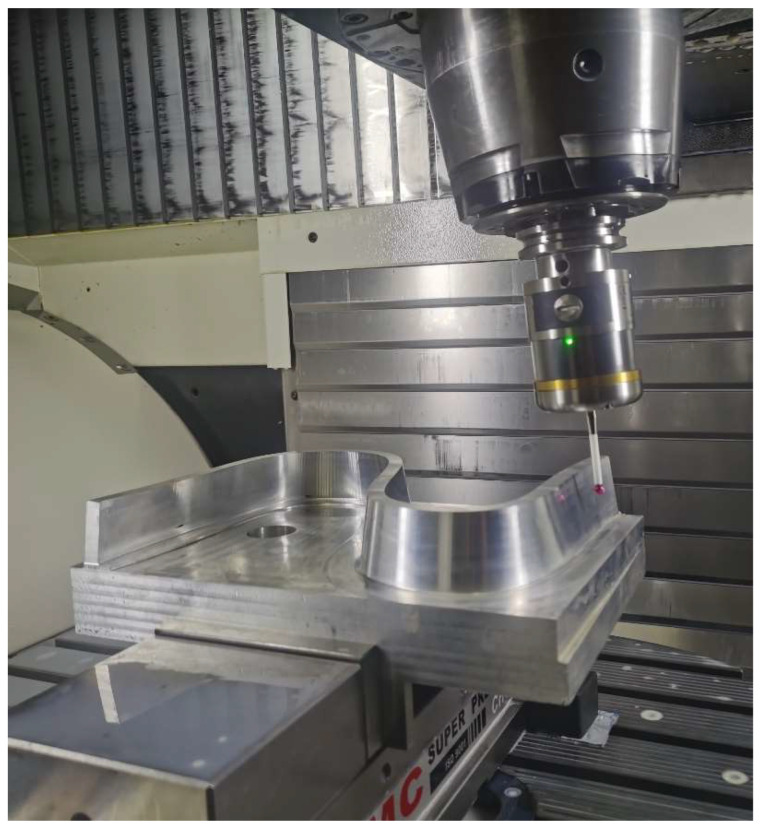
The on-machine verification.

Three S-shaped test pieces were machined using the same cutting parameters, and the NC codes were generated by the CAM software provided by the manufacturers of the machine tool. The inspection experiment was conducted on the self-made on-machine verification with a RENISHAW touch-trigger on-machine probe RMP600. The probe was mounted on the machine spindle and controlled as a tool, so there are no secondary clamping errors during measurement. The on-machine verification is shown in Figure 8. Iso-parametric sampling with a 100 × 8 grid was chosen as the sampling strategy.

### 3.2. Results of the Experiment

Based on the spatial coordinate information given by the probe, the actual points can be expressed as the form in Equation (8). The error mutual moments in each section are calculated according to Equation (9), and the deviations between each corresponding points are calculated according to the following equation:

(12) $$e_{u_{i},v_{i}}=\left\|\vec{P_{u_{i},v_{i}}^{T}P_{u_{i},v_{i}}^{M}}\right\|=\sqrt{{\left(x_{u_{i},v_{i}}^{M}-x_{u_{i},v_{i}}^{T}\right)}^{2}+{\left(y_{u_{i},v_{i}}^{M}-y_{u_{i},v_{i}}^{T}\right)}^{2}+{\left(z_{u_{i},v_{i}}^{M}-z_{u_{i},v_{i}}^{T}\right)}^{2}}$$

**Figure 9:**
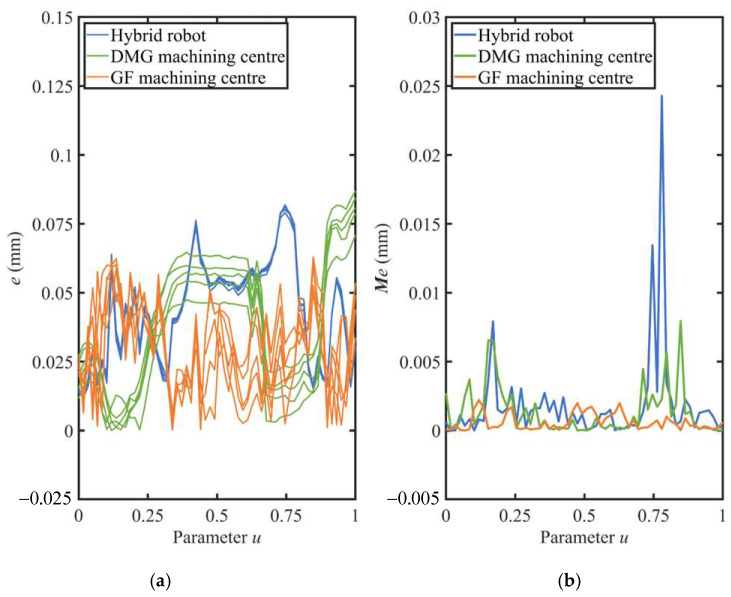
Error distribution of workpieces. (**a**) Normal deviations. (**b**) Error mutual moments.

To demonstrate the superiority of the error mutual moment in assessing the capability for dynamic machining performance of machine tools, the deviations and the error mutual moments of the workpieces were calculated and compared in Figure 9. The error distributions of the workpieces are presented in an increasing order of the parameter *u*. And the data statistics of the deviations and the error mutual moments are provided in Table 5. The maximum value, average value, and the standard deviation are the common indicators that are widely used in engineering applications to assess machining quality. Generally, the maximum value of the deviations is a key indicator of whether the workpiece is qualified or not. The average value of the deviations is a comprehensive indicator to assess machining accuracy. The standard deviation of the deviations is a key indicator to assess consistency in machining accuracy.

Although the error mutual moment and the deviation have the same magnitude, they are on different orders of magnitude. In order to conduct a visual comparison and analysis of the two indicators in assessing the machining performance of the machine tools, it is necessary to deal with the statistical data. In this section, homogenization is applied to accomplish this work, and the equation of homogenization is shown as follows:

(13) $$\left\{\begin{array}{l} Y\left(X_{k}^{Q}\right)=\frac{X_{k}^{Q}}{\bar{X_{k}^{Q}}}\\ \bar{X_{k}^{Q}}=\frac{1}{n}\cdot \sum_{k=1}^{n}X_{k}^{Q} \end{array}\right.,\left\{\begin{array}{l} X=Me,e\\ Q=\mathrm{Maximum}\;\mathrm{value},\;\mathrm{Average}\;\mathrm{value},\;\mathrm{Standard}\;\mathrm{deviation}\;\\ k=1,2,3 \end{array}\right.$$

**Figure 10:**
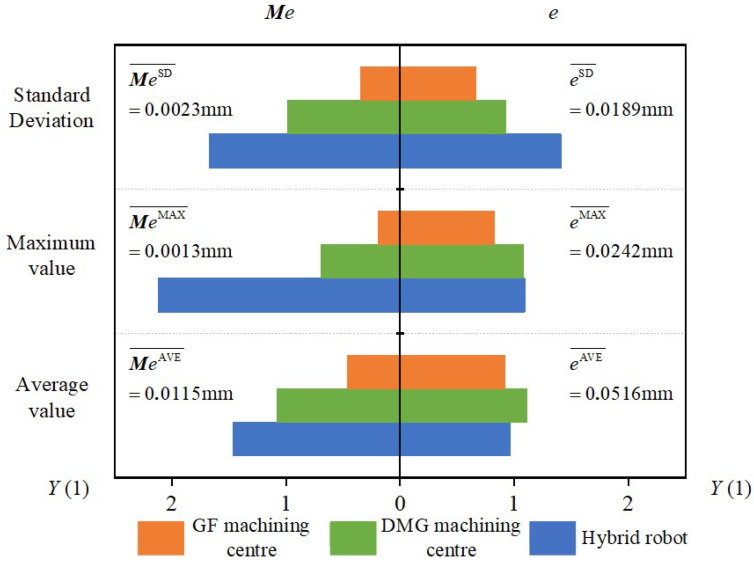
Comparison between error mutual moment and deviation.

Figure 10 illustrates the comparison between the deviation and the error mutual moment following the process of data homogenization.

### 3.3. Discussion

On the one hand, as illustrated in Figure 9, the two indicators are not constant at each section and demonstrate variation with regards to the parameter *u*. Thus, two indicators are correlated with the real-time machining state of the machine tool, including the real-time feedrate, real-time cutting parameter, and other relevant parameters. On the other hand, for a certain machine tool, there is a similar tendency towards both of the two indicators. Nevertheless, for different machine tools, the two indicators show a difference at the same parameter *u*. Thus, the two indicators are correlated with the selection of the machine tool.

Given that the value of the error mutual moment is correlated with the machining state of the machine tool, the error mutual moment can be employed as a target for the optimization of the machining state of the machine tool or of the cutting parameters.

In general, hybrid robots are designed to machine large or super-large parts. Thus, in order to satisfy the demand for high flexibility in hybrid robots, the design and manufacture of these robots have a structure similar to a cantilever beam (shown in Figure 7c). Therefore, with respect to commercial machining centers, hybrid robots demonstrate a weak capability for dynamic machining performance. With regard to the DMG and GF machining centers, both are equipped with a rotatable table. The DMG machining center has the additional rotary axis positioned at the spindle end, while the GF machining center has a cradle-type structure with the additional rotary axis positioned at the table end. Thus, the DMG machining center demonstrates a weak capability for dynamic machining performance compared with the GF machining center. Consequently, it can be empirically demonstrated that the rank of capabilities for dynamic machining performance from weakest to strongest is as follows: hybrid robot, DMG machining center, and GF machining center. In general, for the same cutting parameters, the capability for dynamic machining performance of the machine tool is found to have a negative correlation with the maximum value of the deviation of the machined workpiece. Therefore, empirically, it can be concluded that the maximum deviation of the workpiece, from weakest to strongest, is ranked as follows: GF machining center, DMG machining center, hybrid robot. Empirically based deviation rank is found to correspond with experimental deviation rank. Similarly, the error mutual moment shows the same rank. Furthermore, the error mutual moment shows more significant discrepancies. Therefore, it can be concluded that the error mutual moment can be employed as an additional indicator for assessing the capability for dynamic machining performance of the machine tool, assisting in the determination of the deviation.

## 4. Conclusions and Future Outlook

This paper provides an indicator, namely, the error mutual moment, to assess the capability for dynamic machining performance of the machine tool based on the spatial coordinate information of the touch-trigger on-machine probe considering the characteristic of the error distribution of the flank milling.

1. The characteristic of the error distribution of the flank milling is analyzed and the non-coplanarity between the theoretical and actual contact lines is selected as the basic indicator.
2. A rigorous mathematical derivation is demonstrated for the indicator based on the spatial coordinate information by calculating the degree of non-coplanarity between the theoretical and actual contact lines. The error mutual moment and its model are given.
3. A comparative experiment of the error mutual moment and the deviation is designed, which aims to assess the capability for dynamic machining performance across the machine tools.
4. The results of the experiment proved that the error mutual moment showed more significant difference than the deviation to assess the capability for dynamic machining performance across the machine tools.

The error mutual moment has potential for the optimization of the machining parameters, which leads to the improvement in machining accuracy and machining efficiency. Moreover, the error mutual moment can be applied as a quality assessment sensor for users with a high demand for five-axis machine tools with excellent dynamic machining performance and the manufacturer of machine tools.

## Figures and Tables

**Table 1:** Basic information of the DMG machining center.

Parameters	Values
Numerical control system	HEIDENHAIN TNC530
Ranges of the X/Y/Z axis (mm)	630/560/560
Turning range of B axis	−120°~30°
Rotation range of C axis	0°~360°
Maximum feedrate (m/min)	30

**Table 2:** Basic information of the GF machining center.

Parameters	Values
Numerical control system	HEIDENHAIN TNC640 si
Ranges of the X/Y/Z axis (mm)	500/600/450
Turning range of B axis	−120°~90°
Rotation range of C axis	0°~360°
Maximum feedrate (m/min)	45

**Table 3:** Basic information of the hybrid robot.

Parameters	Values
Numerical control system	Self-made numerical control system
Workspace (mm)	Φ1600 × 400
Maximum feedrate (m/min)	90

**Table 4:** 3D model and the cutting parameters of S-shaped part.

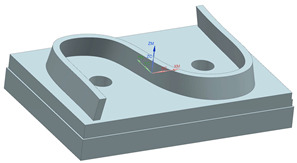	**Cutting Parameters**	**Values**
	Material of the part	Aluminum alloy 7075
	Tool type	End mill
	Tool diameter	20 mm
	Spindle speed	6000 rpm
	Feedrate in finishing	1500 mm/min
	Radial depth of cut in finishing	0.05 mm
	Number of axial cutting layers	1

**Table 5:** Data statistics of the deviations and the error mutual moments.

		DMG Machine Centre	GF Machine Centre	Hybrid Robot
Deviation (mm)	Maximum value	0.0607	0.0464	0.0614
	Average value	0.0269	0.0222	0.0234
	Standard deviation	0.0175	0.0126	0.0267
Error mutual moment (mm)	Maximum value	0.0079	0.0022	0.0243
	Average value	0.0014	0.0006	0.0019
	Standard deviation	0.0023	0.0008	0.0039

## Data Availability

Data are contained within the article.
